# Predictive Measurement of Urethral Mobility for Successful Transurethral Bulkamid Application in Women with Stress Urinary Incontinence

**DOI:** 10.3390/jcm14186555

**Published:** 2025-09-18

**Authors:** Norbert Nosal, Andrea Gerling, Annette Kuhn, Mathieu Pfleiderer, Sunhwa Baek, Sebastian Ludwig

**Affiliations:** 1 Gynecology and Obstetrics, Elisabeth Hospital Essen, 45138 Essen, Germany; 2 Division of Uorgynecology, Inselspital Bern, Women’s Hospital, Switzerland and University of Bern, 3010 Bern, Switzerland; annette.kuhn@insel.ch; 3 University of Cologne, Faculty of Medicine and University Hospital of Cologne, Department of Gynecology and Oncology, Division of Urogynecology, 50937 Cologne, Germany; mathieu.pfleiderer@uk-koeln.de (M.P.); sunhwa.baek@uk-koeln.de (S.B.); sebastian.ludwig@uk-koeln.de (S.L.)

**Keywords:** bulkamid, bulking agent, sonography, ultrasound, urethral mobility

## Abstract

**Background/Objectives**: Bulking agents such as Bulkamid^®^ are well-established surgical options for the treatment of stress urinary incontinence (SUI). Pelvic floor sonographic imaging is readily accessible and may assist in identifying patients who are more likely to benefit from bulking therapies. Urethral mobility appears to significantly influence treatment outcomes and can be classified into hypo-, normo-, and hypermobility. The primary aim of this study was to evaluate the impact of sonographic urethral mobility on the success rate of Bulkamid^®^ injections. The secondary objective was to assess differences between pre- and postoperative urinary incontinence scores. **Methods**: In women with SUI, linear dorsocaudal movement (LDM) of the urethra was measured sonographically. The International Consultation on Incontinence Modular Questionnaire—Urinary Incontinence Short Form (ICIQ-UI SF) was completed prior to Bulkamid^®^ injection. Patients were categorized into hypo-, normo-, and hypermobility groups based on their LDM measurements. **Results**: A total of 130 patients participated, with 101 undergoing both pre- and postoperative sonographic assessment. The difference in LDM before and after treatment was calculated. Patients with normomobile urethras (n = 79) exhibited the greatest mean improvement in continence scores, with LDM changes ranging from 6 to 24 mm and an average ICIQ-UI SF score reduction of 3.8 points. Patients with hypomobile (n = 16) or hypermobile urethras (n = 6) also demonstrated improvements, but to a lesser extent than the normomobile group. **Conclusions**: This study indicates that patients with a normomobile urethra experience the most significant improvement in continence outcomes following Bulkamid^®^ injection. Urethral mobility assessment via sonography may serve as a valuable preoperative tool and appears to play a crucial role in predicting treatment success with bulking agents.

## 1. Introduction

Urinary incontinence (UI) is characterized by the involuntary loss of urine resulting from a diminished or absent capacity of the body to consciously retain bladder contents. Among the various forms of UI, stress urinary incontinence (SUI) is one of the most prevalent, affecting approximately 7.8% of women aged 18 to 40 years and increasing to 27.1% in women over 60 years of age [1]. In addition to conservative management strategies such as pelvic floor muscle training and continence aids, surgical interventions have gained increasing popularity in recent years [1]. Several surgical options exist for the treatment of SUI in women, with tension-free vaginal tape (TVT) being regarded as the current gold standard [2]. Multiple studies have demonstrated that Bulkamid^®^ exhibits a success rate exceeding 70% at five years post-treatment, with outcomes typically categorized into “cured” (17% to 47%) and “improved” (23% to 47%) [3,4].

Pelvic floor sonography serves as a valuable tool both preoperatively, for identifying patients less likely to respond to treatment, and postoperatively, for visualizing the distribution of bulking agent deposits [5,6]. Comparing pre- and postoperative sonographic assessments alongside objective measures of continence improvement provides robust evidence regarding the efficacy of continence surgery. Notably, in more than 40% of cases, deviations from the ideal or target injection site—approximately 1.5 cm caudal to the internal urethral orifice—are observed during postoperative sonographic evaluation [7]. Preoperative sonographic assessment of urethral mobility and length appears to be a superior predictor of the postoperative localization of Bulkamid^®^ deposits and their dynamic effects [8].

Further, preoperative measurement of urethral mobility via sonography has been shown to predict postoperative continence outcomes [6,9]. However, such assessments have not yet been extensively explored in the context of Bulkamid^®^ therapy. The current classification of urethral mobility—namely hypo-, normo-, and hypermobility—is primarily derived from studies involving other surgical procedures such as TVT and colposuspension, and has not been specifically applied in conjunction with bulking agents. The aim of this study was to investigate the impact of a novel classification based on linear dorsocaudal movement (LDM) of the urethra on postoperative urinary continence function.

## 2. Materials and Methods

Between 2018 and 2019, bulking therapy with Bulkamid^®^ was administered to a cohort of 130 women diagnosed with stress urinary incontinence (SUI) by two experienced surgeons (>20 procedures per surgeon) at Elisabeth Hospital Essen, Germany. This research was conducted as a retrospective case–control study.

**Figure 1:**
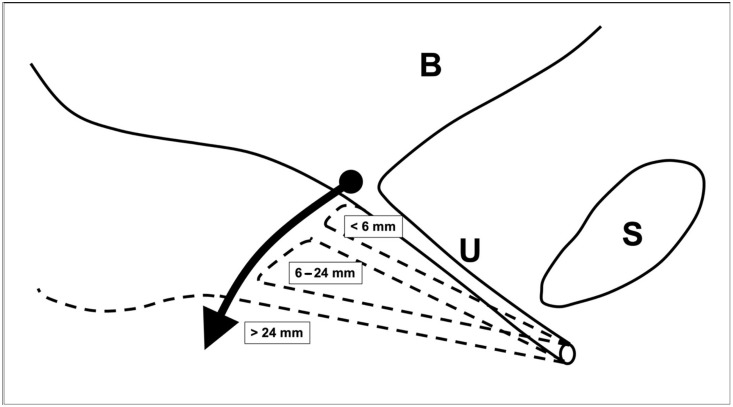
Schematic illustration of urethral mobility assessed through linear dorsocaudal movement (LDM). The arrow shows an increase of urethral mobility. The measurement points were the edge of the symphysis pubis and the internal urethral orifice. Hypomobility is defined as urethral mobility—specifically, vertical displacement—of less than 6 mm (<6 mm), normomobility corresponds to displacement between 6 mm and 24 mm (6–24 mm), and hypermobility is characterized by displacement exceeding 24 mm (>24 mm) [6].

All participants presenting to the urogynecology department completed the validated International Consultation on Incontinence Modular Questionnaire—Urinary Incontinence Short Form (ICIQ-UI SF) [10], along with a self-developed questionnaire concerning urinary incontinence history. Preoperative pelvic floor sonography was performed by attending physicians and subsequent surgeons with patients in a semi-seated position on a gynecological chair in lithotomy. Imaging utilized a 2D vaginal probe (GE^®^, model Voluson E8, Wien, Austria) with a frequency range of 3.5–10 MHz. Measurements were standardized relative to the upper edge of the symphysis pubis using horizontal and vertical reference lines, with the distance to the bladder neck recorded in millimeters perpendicular to the bladder axis [6]. The probe was held aligned at zero degrees to the vaginal axis (see Figure 1).

Urethral mobility was assessed by measuring the sonographic displacement of the bladder neck—specifically, the change in position (“in mm”) from rest to Valsalva maneuver with an empty bladder to gauge the dorsocaudal movement (LDM) [6]. During the Valsalva, patients remained seated in lithotomy position, instructed to perform maximal abdominal and trunk muscle contractions without internal or external straining for at least ten seconds. For introitus sonography, a GE^®^ vaginal transducer (model Intercavity Array Ultrasound IC9-RS, Wien, Austria) was employed with minimal applied pressure.

Urethral mobility classifications were based on prior literature, with the traditional grouping as follows: hypomobility (<6 mm LDM), normomobility (6–15 mm LDM), and hypermobility (>15 mm LDM) [6,8,11]. For this study, classifications from Wlazlak et al. (hypomobility: LDM < 6 mm) and Naranjo-Ortiz and Dietz (hypermobility: LDM > 24 mm) were integrated into a “physiological classification,” defining mobility as: hypomobility (<6 mm), normomobility (6–24 mm), and hypermobility (>24 mm) [11].

**Figure 2:**
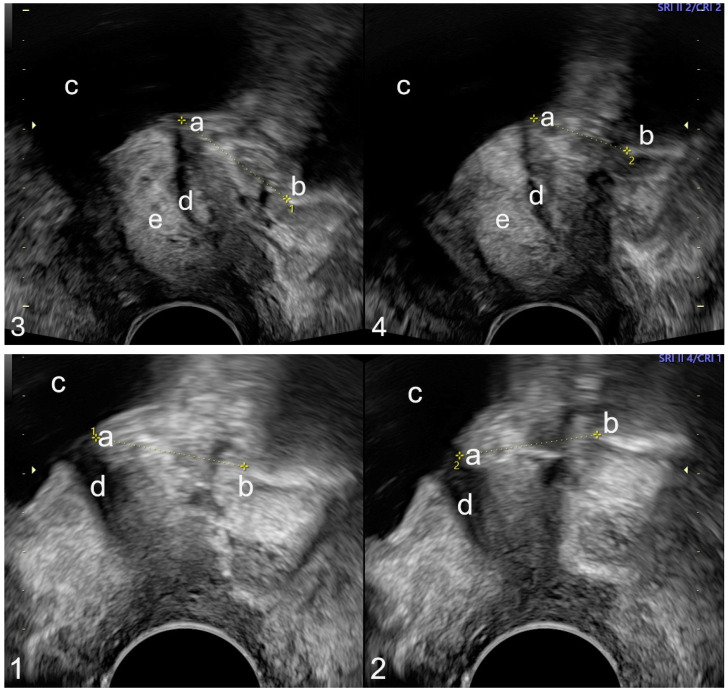
Pelvic floor sonography was performed using a 2D vaginal probe with a frequency range of 3.5–10 MHz (GE^®^, model Voluson E8, Wien, Austria), targeting the internal urethral orifice (a), symphysis pubis edge (b), bladder (c), and urethra (d). Imaging was conducted preoperatively (1,2) and postoperatively (3,4) to assess urethral mobility via linear dorsocaudal movement (LDM, yellow dashed line), utilizing the hyperechoic Bulkamid^®^ (e) three days after Bulkamid^®^ injection. Specifically: (1) preoperative at rest (LDM 19 mm), (2) preoperative under stress (LDM 18 mm), (3) postoperative at rest (LDM 20 mm), and (4) postoperative under stress (LDM 16 mm).

Patients received comprehensive counseling regarding treatment options; subsequent intervention decisions were based on patient preferences [12]. Blinding was not applied, and surgical intervention was indicated solely by demonstrable urine leakage during the Valsalva maneuver. Bulkamid^®^ injections were performed under either general or local anesthesia, following preoperative assessment for urinary tract infections (via dipstick testing) and perioperative prophylaxis with a second-generation cephalosporin IV. A standardized transurethral injection of 2 mL Bulkamid^®^ (Contura^®^) was delivered into four quadrants (0.5 mL each) using a 22G, 120 mm needle and a urethrocystoscope. Depots were then sonographically examined in two orthogonal planes postoperatively to confirm correct placement or identify dislocated or insufficient deposits, aiming to achieve a two-dimensional sealing ring around the urethra (see Figure 2).

Follow-up assessments occurred between nine and twelve months post-injection, utilizing the ICIQ-UI SF questionnaire without blinding by clinicians. For cases with inadequate subjective response, patients were offered a repeat Bulkamid^®^ injection (Re-Bulkamid^®^), with pre-reinjection sonographic depots measured similarly to evaluate depot integrity and volume adequacy. The Clavien–Dindo classification was employed to document complications [13].

**Figure 3:**
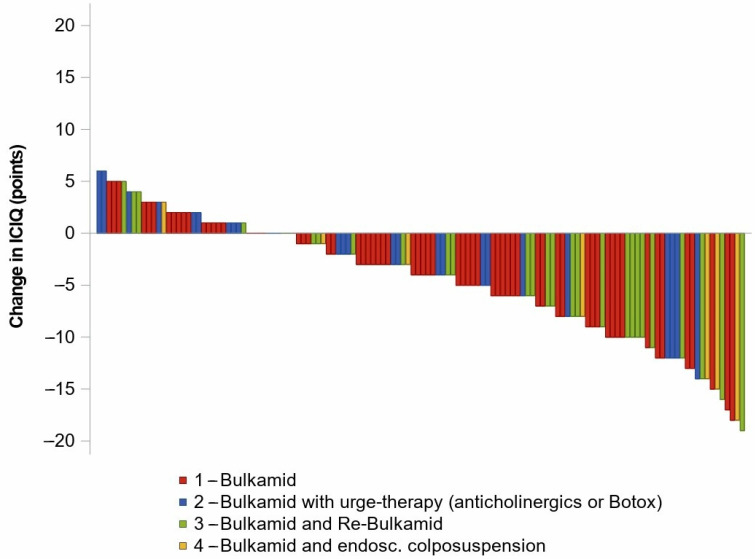
Individual absolute changes in ICIQ-UI SF scores among 130 women with urinary incontinence were assessed 9–12 months post-surgery. The waterfall diagram displays the change in ICIQ-UI SF points (y-axis) for each of the 130 patients (x-axis). Each column represents the difference in ICIQ-UI SF scores before and after treatment. The data are categorized into four groups based on treatment modality: (1) single Bulkamid^®^ application (red), (2) Bulkamid^®^ in combination with urge therapy, including anticholinergics or botulinum toxin (blue), (3) Bulkamid^®^ with Re-Bulkamid^®^ (green), and (4) Bulkamid^®^ with subsequent endoscopic colposuspension (laparoscopic Burch procedure) (yellow). Re-Bulkamid^®^ refers to a repeat Bulkamid^®^ injection. A positive difference to the left indicates an increase in incontinence severity after surgery, whereas a negative difference to the right indicates an improvement with reduced incontinence.

Data analysis involved univariate statistical methods using SAS 9.4 software, focusing on descriptive statistics such as means, medians, and confidence intervals (CIs) for each treatment group (see Table 1). Changes in ICIQ-UI SF scores pre- and post-treatment, including absolute and relative differences with CIs, were calculated (see Table 2). Outcomes were stratified by urethral mobility groups—hypomobile, normomobile, and hypermobile—based on the sonographic LDM measurements (see Table 3). Additionally, a waterfall plot illustrating individual patient changes in ICIQ-UI SF scores, color-coded according to mobility group, was generated using SAS 9.4 (see Figure 3).

## 3. Results

A total of 130 patients treated with Bulkamid^®^ completed the ICIQ-UI SF questionnaire between nine and twelve months following their last injection. Table 1 summarizes the demographic and clinical characteristics of the cohort. The median age was 69 years, with the first quartile (Q1) at 61 years and the third quartile (Q3) at 79 years. Considering that an ICIQ-UI score exceeding 11 indicates severe urinary incontinence (UI), the majority of participants in this study presented with severe symptoms, evidenced by a mean preoperative ICIQ-UI score of 14.7 (SD ± 3.5). The median sonographically measured urethral length was 28 mm (range 27–31 mm), while the median urethral mobility was 11 mm (range 6–17 mm). The median operative duration was 9 min (range 6–13 min). Twenty-two percent (n = 28) of the procedures were performed under local anesthesia. Thirteen patients had previously undergone tension-free vaginal tape (TVT) procedures, with the tape either still in situ or removed prior to Bulkamid^®^ therapy.

Table 2 details the changes in ICIQ-UI scores following treatment across different surgical groups. A single Bulkamid^®^ application was sufficient in 67 of the 130 patients (52%). The median age within this subset was 69 years (Q1: 63; Q3: 74). The mean preoperative ICIQ-UI score was 14.1 (95% CI: 13.2–14.9), which decreased to 9.9 (95% CI: 8.6–11.2) postoperatively. Twenty-one percent (n = 28) of patients required additional treatment for urgency urinary incontinence (UUI), such as anticholinergic medication or botulinum toxin injections. Their median age was 71 years (Q1: 66; Q3: 80), with pre- and postoperative ICIQ-UI scores of 14.9 (95% CI: 13.6–16.2) and 12.4 (95% CI: 10.0–14.7), respectively. Re-Bulkamid^®^ therapy was performed in 28 patients (22%), with a median age of 74 years (Q1: 64; Q3: 79). In this group, the preoperative ICIQ-UI score was 15.4 (95% CI: 13.9–17.0), decreasing to 9.6 (95% CI: 7.3–11.9) after re-injection. Eight patients (6%) underwent surgical conversion to endoscopic colposuspension (EC), with a median age of 56 years (Q1: 58; Q3: 63). Their preoperative ICIQ-UI score was 16.0 (95% CI: 13.7–18.3), which reduced to 9.0 (95% CI: 3.5–14.5) postoperatively. The mean reduction in ICIQ-UI score among women receiving only one Bulkamid^®^ injection was −4.1 points (95% CI: −5.5 to −2.8). Patients undergoing Re-Bulkamid^®^ experienced an initial score reduction of −3.3 points (95% CI: −5.4 to −1.2) after the first injection, with a total improvement of −5.6 points (95% CI: −8.2 to −3.5) after both procedures. Notably, this group demonstrated a higher baseline ICIQ-UI score (15.4; 95% CI: 13.9–17.0) compared to those treated with Bulkamid^®^ alone (14.1; 95% CI: 13.2–14.9) (see Table 2).

**Figure 4:**
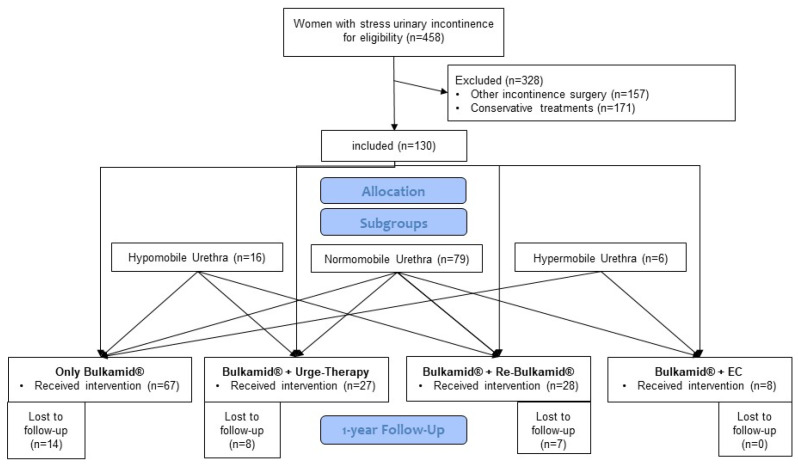
Flowchart of Department Urogynecology at Elisabeth Hospital Essen, Germany, from 2018–2019 about 130 treatments with Bulkamid^®^. The data are categorized into four groups based on treatment modality: Single Bulkamid^®^ application, Bulkamid^®^ in combination with urge therapy, Bulkamid^®^ with Re-Bulkamid^®^ and Bulkamid^®^ with subsequent endoscopic colposuspension (EC, laparoscopic Burch procedure). Re-Bulkamid^®^ refers to a repeat Bulkamid^®^ injection.

Table 3 delineates the changes in ICIQ-UI scores as an indicator of continence following Bulkamid^®^ and Re-Bulkamid^®^ interventions. Due to the refusal of postoperative ultrasound examination by 29 of the 130 patients (22%), the analysis was performed on data from 101 patients (see Figure 4). Patients with hypomobile urethras exhibited a mean change of −0.8 (95% CI: −2.7; 1.1) in ICIQ-UI score after Bulkamid^®^, with no significant alteration observed after Re-Bulkamid^®^ (−0.8; 95% CI: −6.5; 4.9). Patients with normomobility showed a mean reduction of −3.8 (95% CI: −5.0; −2.5) following Bulkamid^®^ and a further reduction of −6.3 (95% CI: −8.7; −3.9) after Re-Bulkamid^®^. In contrast, hypermobile patients experienced a change of −2.5 (95% CI: −7.7; 2.7) after Bulkamid^®^. Both pathological mobility groups—hypomobile and hypermobile—exhibited lower ICIQ-UI scores after treatment with Bulkamid^®^ and Re-Bulkamid^®^ compared to normomobile patients. Overall, 70 of the 130 patients (54%) reported subjective improvement, while an additional 19 patients (15%) achieved a cure. Transient postoperative urinary retention was observed in 6 patients (4%) and was managed successfully with disposable 8-Charrière catheters, resolving within one day (Clavien–Dindo grade I). Postoperative urinary tract infections occurred in eleven patients (8%) and were treated effectively with oral antibiotics (Clavien–Dindo grade II).

## 4. Discussion

To date, limited data exist regarding the predictive value of preoperative sonographic assessment of urethral mobility for clinical outcomes following Bulkamid^®^ therapy. The current classification of urethral mobility—hypomobility, normomobility, and hypermobility—may influence treatment recommendations, potentially limiting the use of Bulkamid^®^ in cases of hypermobility, particularly according to previous definitions. Notably, hypomobility defined by a urethral displacement of less than 6 mm has not yet been described in relation to Bulkamid^®^ treatment [1,2,8].

Our findings support the hypothesis that preoperative ultrasound measurement of urethral mobility could serve as a predictor of postoperative success and provide valuable information for preoperative counseling. In our cohort, patients classified within the normomobile range (6–24 mm) exhibited the most favorable outcomes following Bulkamid^®^ treatment. The smallest reduction in ICIQ-UI scores was observed in the hypomobile group (<6 mm), whereas the hypermobile group (>24 mm) demonstrated better results than the hypomobile group, although the outcomes did not reach the level observed in the normomobile cohort.

In a study by Giammò et al., urethral mobility was not assessed via ultrasound but using the Q-tip test to measure the angle of rotation during the Valsalva maneuver [14]. This method defines urethral hypermobility at an angle exceeding 28 degrees during straining. However, the Q-tip approach has been shown to have limited predictive value regarding clinical outcomes related to urinary incontinence [14]. The classification based on this method corresponds to a sonographic measurement (LDM) threshold of 14.6 mm for hypermobility [6], although the Q-tip technique is somewhat invasive and less practical in routine clinical settings.

For a more accurate and feasible assessment in the context of Bulkamid^®^ treatment, we utilized ultrasound measurements. Naranjo-Ortiz and Dietz established a hypermobility cutoff of >24 mm, aligning with the physiological classification used in this study [5,11,15]. This threshold corresponds to the traditional classification derived from TVT and colposuspension procedures for SUI [9].

Furthermore, the International Continence Society (ICS) recommends assessing bladder neck mobility preoperatively in cases of hypomobility [16], to evaluate the potential role of fillers in cases where intrinsic sphincter deficiency (ISD) persists [17]. Postoperative continence rates tend to be lower with other surgical interventions, such as TVT [8], yet many clinicians continue to advocate for the use of fillers in patients exhibiting hypomobility [16,18].

One possible explanation for the observed results lies in our stratification of urethral mobility into hypo-, normo-, and hypermobility categories. The previous classification, which designated hypermobility at a threshold of 14.6 mm, was found to be insufficient for predicting the efficacy of Bulkamid^®^ treatment, as mechanical compression of the urethra in its lower half did not correlate with clinical outcomes. The therapeutic effect is likely attributable to coaptation of the gaping urethral mucosa [19], a potential enhancement of urethral sphincter strength [20], and consequently an increase in urethral sphincter resistance [21].

The pathogenesis of urinary leakage varies between hypermobility and hypomobility. While hypermobility is frequently associated with damage to the pubourethral ligaments [22], making it amenable to surgical corrections such as TVT and colposuspension [1], hypomobility is often linked with intrinsic sphincter deficiency (ISD), comprising urethral sphincter weakness [17]. Our study did not specifically address Bulkamid^®^ use in this subgroup. Notably, the postoperative reduction in the ICIQ-UI SF score was least pronounced within this group, and even Re-Bulkamid^®^ did not achieve significant improvement, although the number of cases was limited.

In accordance with German guidelines [1], we did not perform urodynamic assessments in all patients. However, 23 out of 130 patients underwent urodynamic testing when clinical suspicion of severe stress urinary incontinence was high. These examinations revealed an abdominal leak point pressure (ALPP) below 60 cm H_2_O, indicative of severe SUI.

Our findings suggest that preoperative measurement of urethral mobility can serve as an initial indicator for surgical decision-making and may provide additional insights into expected treatment success. Patients with hypomobility should be counseled regarding the reduced likelihood of postoperative success, even with Re-Bulkamid^®^. Furthermore, a higher baseline ICIQ-UI SF score appears to predict a greater necessity for reinjection. Age was generally not strongly correlated with treatment choice; median age for Bulkamid^®^ was 69 years (range 63–74), for Re-Bulkamid^®^ 74 years (range 64–79), whereas patients undergoing endoscopic colposuspension (EC) were significantly younger, with a median of 56 years (range 58–63).

This study lends support to our understanding of patient selection criteria for successful outcomes. The strengths include the relatively large sample size and the standardized approach to surgical procedure. The number of surgeons experienced in both sonography and Bulkamid^®^ injection was small, which reduced variability. However, limitations include the retrospective nature of the study, potential detection bias due to the lack of blinding, and a follow-up rate of 78%, which may introduce bias if non-responders experienced different outcomes. Additionally, confounding variables such as BMI, parity, and menopausal status were not controlled, and there was no standardized objective measurement of intra-abdominal pressure during the Valsalva maneuver, which could have influenced the classification and results. The small number of patients with hypomobility (n = 16) and hypermobility (n = 6) may limit the statistical power, underscoring the need for future prospective studies with larger cohorts and objective measures such as cough tests, pad tests, or urodynamics.

## 5. Conclusions

In the context of a retrospective cohort study, the findings offer preliminary evidence that preoperative sonographic risk profiles may be associated with the outcomes of Bulkamid^®^ treatment. Subsequently, a prospective, multicenter, randomized trial should be conducted, incorporating 2 mL Bulkamid^®^ injections and 3 mL injections (including Re-Bulkamid^®^), with preoperative sonographic classification into hypo-, normo-, and hypermobility. Additionally, objective measures should be integrated to establish this assessment as a potential standard preoperative evaluation tool.

## Figures and Tables

**Table 1:** Characteristics of the 130 patients who underwent surgical treatment for urinary incontinence with Bulkamid^®^ and Re-Bulkamid^®^.

		Post Hoc Subgroups			
	Overall Cohort (n = 130)	Bulkamid (n = 67)	Urge-Therapy (n = 27)	Re-Bulkamid (n = 28)	Endoscopic Colposuspension (n = 8)
Age in years (median, Q1, Q3)	69 (61; 77)	69 (63; 74)	71 (66; 80)	73.5 (64; 79)	56 (48; 63)
Preoperative ICIQ-UI SF (mean, SD)	14.7 (SD 11.2–18.2)	14.1 (10.7; 17.5)	14.9 (11.5; 18.3)	15.4 (11.4; 19.4)	16.0 (13.2; 18.8)
Urethra length in mm (median, Q1, Q3)	28 (26; 31)	28 (27; 31)	29 (26; 30)	28.5 (26.5; 30.5)	28.5 (26.5; 30.5)
Urethra mobility in mm (median, Q1, Q3) (n = 107)	11 (6; 17)	8.5 (7.0; 13.5)	11 (4; 20)	10 (6; 16)	24 (15; 30)
Pretreated with TVT (n)	13	5	2	5	1
OP-time (min) (median, Q1, Q3)	9 (7; 10)	9 (7; 10)	8.5 (7; 11)	9 (7; 11)	8 (7; 11)
Anesthetics					
Local (n)	29	15	6	6	1
General (n)	108	52	21	22	7

ICIQ-UI SF, International Consultation on Incontinence Questionnaire-Urinary Incontinence short form; TVT, tension-free vaginal tape.

**Table 2:** Mean ICIQ-UI score changes recorded preoperatively and up to 12 months postoperatively (following Bulkamid^®^ and Re-Bulkamid^®^ treatments) for the 130 women with urinary incontinence. Re-Bulkamid^®^ indicates a second injection of Bulkamid^®^.

Type of Surgery	Time Points of Measurement	n	ICIQ-UI SF Score Mean Value (95%CI)	Absolute Difference from Initial Value (95% CI)	Relative Difference from Initial Value (95% CI)
Bulkamid	before Bulkamid	67	14.1 (13.2; 14.9)	-	-
	after Bulkamid	67	9.9 (8.6; 11.2)	−4.1 (−5.5; −2.8)	−0.28 (−0.38; −0.18)
Re-Bulkamid	before Bulkamid	28	15.4 (13.9; 17.0)	-	-
	after Bulkamid	28	12.1 (10.1; 14.2)	−3.3 (−5.4; −1.2)	−0.19 (−0.33; −0.05)
	after Re-Therapy	28	9.6 (7.3; 11.9)	−5.6 (−8.2; −3.5)	−0.36 (−0.52; −0.21)

ICIQ-UI SF, International Consultation on Incontinence Questionnaire-Urinary Incontinence short form; For better illustration, the two groups “urge therapy” (n = 27) and “endoscopic colposuspension” (n = 8) were hidden.

**Table 3:** ICIQ-UI SF score outcomes (cured; improved) in relation to sonographically measured urethral mobility following Bulkamid^®^ and Re-Bulkamid^®^ therapy among 130 women with urinary incontinence.

Mobility	n (Total)	Cured (n)	ICIQ-UI SF Score Change After Bulkamid^®^ (95% CI)	Improved (n)	n (After Re-Bulkamid^®^)	ICIQ-UI SF Score Change After Re-Bulkamid^®^ (95% CI)
Hypomobile (<6 mm)	16	0	−0.8 (−2.7; 1.1)	8	5	−0.8 (−6.5; 4.9)
Normomobile (6–24 mm)	79	13	−3.8 (−5.0; −2.5)	41	28	−6.3 (−8.7; −3.9)
Hypermobile (>24 mm)	6	0	−2.5 (−7.7; 2.7)	3	2	−4.5 (−62.7; 51.7)
Lost for follow-up	29	-	-	-	-	-

ICIQ-UI SF, International Consultation on Incontinence Questionnaire-Urinary Incontinence short form Twenty-nine of the 130 patients (22%) refused further postoperative sonography examination.

## Data Availability

The datasets used and analyzed during the current study are not publicly available because they contain sensitive patient data, but they are available from the corresponding author in an anonymized and de-identified form upon reasonable request.

## Abbreviations

The following abbreviations are used in this manuscript:

AEKNO	North Rhine Westphalia Medical Association
ALPP	Abdominal leak point pressure
CI	Confidence interval
EC	Endoscopic colposuspension
ICIQ-UI SF	International Consultation on Incontinence Modular Questionnaire Urinary Incontinence short form
LDM	Linear dorsocaudal movement
SUI	Stress urinary incontinence
TVT	Tension-free vaginal tape
UI	Urinary incontinence
